# Maternal occupational exposures and fetal growth in a Spanish birth cohort

**DOI:** 10.1371/journal.pone.0264530

**Published:** 2022-04-07

**Authors:** Jennifer Ish, David Gimeno Ruiz de Porras, Elaine Symanski, Ferran Ballester, Maribel Casas, George L. Delclos, Mònica Guxens, Jesús Ibarluzea, Carmen Iñiguez, Loreto Santa-Marina, Michael D. Swartz, Kristina W. Whitworth

**Affiliations:** 1 Southwest Center for Occupational and Environmental Health (SWCOEH), Department of Epidemiology, Human Genetics, and Environmental Sciences, The University of Texas Health Science Center at Houston (UTHealth) School of Public Health in San Antonio, San Antonio, TX, United States of America; 2 Department of Medicine, Baylor College of Medicine, Houston, TX, United States of America; 3 Center for Precision Environmental Health, Baylor College of Medicine, Houston, TX, United States of America; 4 CIBER Epidemiología y Salud Pública, Madrid, Spain; 5 Department of Nursing, Universitat de València, Valencia, Spain; 6 Epidemiology and Environmental Health Joint Research Unit, FISABIO–Universitat Jaume I Universitat de València, València, Spain; 7 Universitat Pompeu Fabra, Barcelona, Spain; 8 ISGlobal, Barcelona, Spain; 9 Center for Research in Occupational Health (CiSAL), Universitat Pompeu Fabra, Barcelona, Spain; 10 Southwest Center for Occupational and Environmental Health (SWCOEH), Department of Epidemiology, Human Genetics, and Environmental Sciences, UTHealth School of Public Health, Houston, TX, United States of America; 11 Department of Child and Adolescent Psychiatry, Erasmus MC, University Medical Centre, Rotterdam, The Netherlands; 12 Environmental Epidemiology and Child Development Group, Biodonostia, San Sebastian, Spain; 13 Health Department of the Basque Government, Sub-directorate of Public Health of Gipuzkoa, San Sebastian, Spain; 14 Faculty of Psychology, Universidad del País Vasco (UPV/EHU), San Sebastian, Spain; 15 Department of Statistics and Operational Research, Universitat de València, València, Spain; 16 Department of Biostatistics and Data Science, UTHealth School of Public Health, Houston, TX, United States of America; Tulane University School of Public Health and Tropical Medicine, UNITED STATES

## Abstract

While the epidemiologic literature suggests certain maternal occupational exposures may be associated with reduced measures of size at birth, the occupational literature employing fetal biometry data to assess fetal growth is sparse. The present study examines associations between maternal occupational exposures and ultrasound-measured fetal growth. We included 1,739 singleton pregnancies from the *INfancia y Medio Ambiente* (INMA) project (2003-2008). At 32 weeks of pregnancy, interviewers ascertained mothers’ employment status and assessed job-related physical loads, work schedules, and job strain during pregnancy. Job titles were linked to a job-exposure matrix to estimate exposure to 10 endocrine disrupting chemical (EDC) groups. We calculated z-scores from longitudinal growth curves representing trajectories from 0-12, 12-20 and 20-34 gestational weeks for abdominal circumference (AC), biparietal diameter (BPD), femur length (FL), and estimated fetal weight (EFW). Linear mixed models clustered by IMNA region (i.e., Gipuzkoa, Sabadell, Valencia) were used to examine associations between occupational exposures and fetal growth. Effect estimates are presented as percentage change in fetal growth. There was limited evidence of associations between work-related non-chemical stressors and fetal growth. We observed associations of similar magnitude between multiple EDC groups and decreased EFW trajectories during 20-34 gestational weeks (phthalates: -1.4% [-3.5, 0.6%]; alkylphenolic compounds (APCs): -1.1% [-2.3, 0.1%]; miscellaneous chemicals: -1.5% [-3.7, 0.8%]), while miscellaneous chemicals were associated with increased BPD from 12-20 weeks (2.1% [0.8, 3.5%]). Notably, 67% of women exposed to phthalates were hairdressers; 68% of women exposed to APCs worked as domestic cleaners. In conclusion, we found limited evidence that maternal occupational exposures impact fetal growth. Further research should consider the combined impact of multiple workplace exposures.

## Introduction

In European Union countries, approximately three-fourths of adult women participate in the workforce and most are in their reproductive years [1]. Thus, a significant portion of pregnant women are at risk of exposure to reproductive and developmental hazards at work. During pregnancy, the fetus is particularly vulnerable to environmental insults [2], and exposures that alter fetal growth may predispose individuals to increased risk of morbidity and mortality throughout the life-course [3,4].

An increasing number of epidemiologic studies link environmental endocrine disrupting chemical (EDC) exposure among pregnant women to reduced measures of fetal growth [5,6], yet limited data exist in relation to maternal exposure to EDCs in occupational settings. Women working in certain professions may be exposed to higher concentrations or come into more frequent contact with EDCs than the community at large [7,8], thereby potentially leading to fetal exposure at levels above background concentrations. Available literature suggests that mothers who are occupationally exposed to EDCs—in particular, organic solvents, pesticides, and phthalates—are more likely to have infants who are born low birth weight (LBW; birthweight <2,500 g) or small for gestational age (SGA; birthweight in <10^th^ percentile for gestational age) [9–11]. However, measures at birth do not capture the dynamic nature of fetal growth; it is possible that restricted growth occurs during pregnancy even if the fetus achieves population size standards at birth [12]. Further, anthropometric measures at birth poorly reflect growth trajectories during early pregnancy, which may be the most relevant period for susceptibility to external stressors and subsequent health effects [13]. Only one study to date has investigated whether ultrasound-measured fetal growth is sensitive to maternal occupational EDC exposures, finding that exposure to several chemicals, including polycyclic aromatic hydrocarbons (PAHs), phthalates, alkylphenolic compounds (APCs) and pesticides, were associated with reduced trajectories of fetal length, head circumference, and estimated fetal weight [14].

In addition to chemical exposures, women may be exposed to job-related non-chemical stressors that pose a potential risk to fetal growth [15–17]. In the Sixth European Working Conditions Survey [18], 23% of women report carrying heavy loads, 21% report engaging in shift work, and 14% report working night shifts. Many women also experience conditions that contribute to psychosocial work stress, including the inability to influence decisions that are important for their work (29%), performing monotonous tasks (45%), and low levels of support from supervisors and co-workers (18% and 11%, respectively). Two recent meta-analyses found that several occupational exposures, including prolonged standing, heavy lifting, rotating shift work, and night work, may be associated with LBW or SGA, but evidence is inconclusive [16,17]. Thus, further research is needed to examine the potential impact of non-chemical occupational stressors on fetal biometry, which may provide insight on how physically demanding work contributes to adverse birth outcomes. To date, one study has been conducted to this end. While the authors found no consistent associations between prolonged standing or heavy lifting (≥25 kg) with SGA or LBW, they found statistically significant associations between these metrics and ultrasound-based measures of fetal growth, highlighting the importance of examining size during fetal life [19]. In relation to occupational psychosocial stress or shift work, to our knowledge, fetal growth trajectories have not been evaluated in the previous literature.

The objective of the present study is to examine whether maternal exposures to several classes of EDCs and various non-chemical workplace stressors influence fetal growth among mother-child pairs in a prospective birth cohort. We hypothesize that maternal occupational exposure to physical and psychosocial stressors and EDCs will be associated with reduced fetal growth trajectories.

## Materials and methods

### Study population

**Fig 1:**
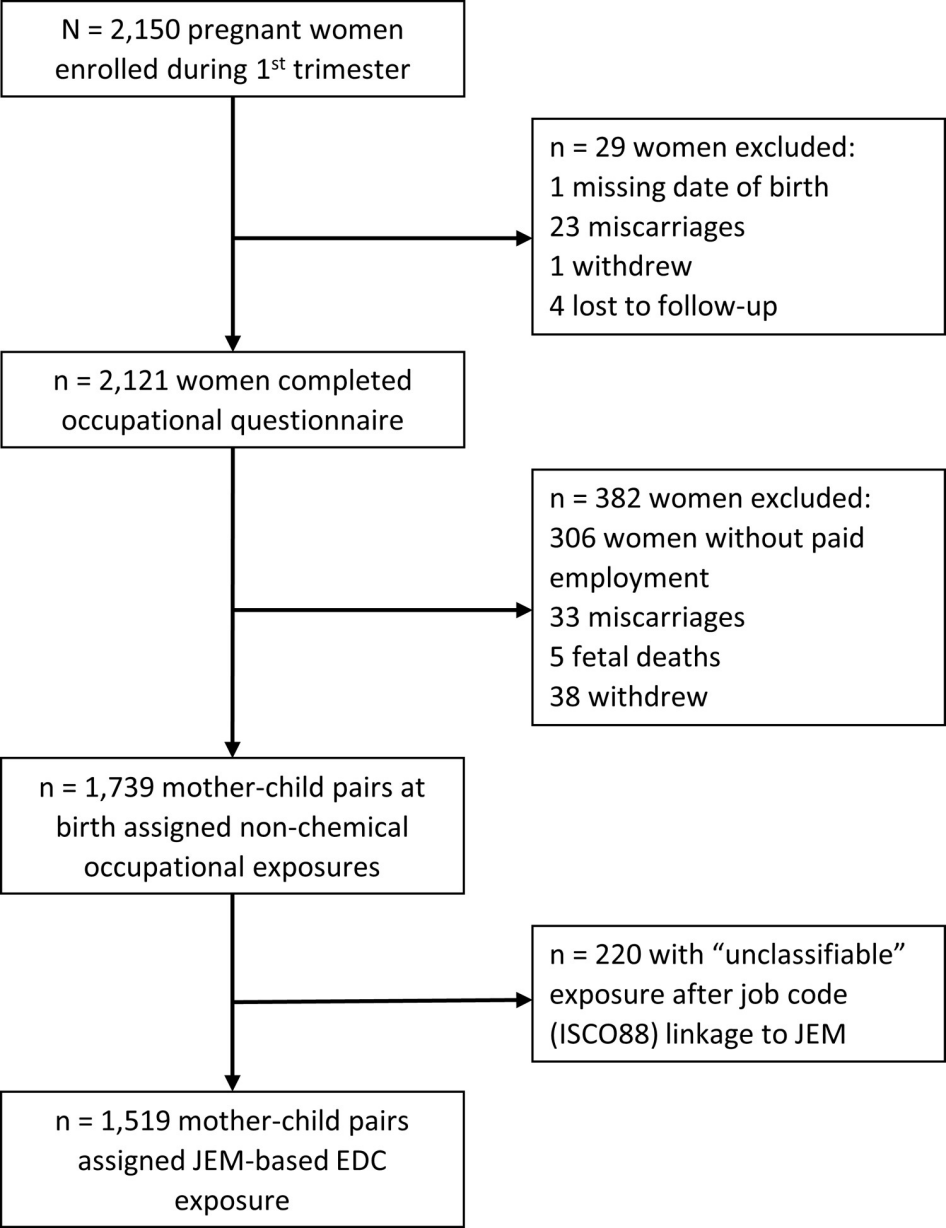
Study flowchart for INMA participants in the Gipuzkoa, Sabadell and Valencia cohorts, 2003-2008 (Spain). 1.

The INMA Project (*INfancia y Medio Ambiente*; Childhood and Environment) is a network of population-based prospective birth cohorts in Spain [20]. The present analysis is based on data from three INMA regions: Gipuzkoa, Sabadell, and Valencia. In each region, recruitment took place at the main public hospital between 2003 and 2008. Women were recruited at their first routine prenatal care visit if they met the following eligibility criteria: ≥16 years of age, singleton pregnancy, enrollment at 10-13 weeks of gestation, non-assisted conception, intention to deliver at reference hospital, and no communication impairment. The INMA Project was approved by the Clinical Research Ethics Committees of the University Hospital of La Fe in Valencia (Valencia, Spain), Donostia Hospital (San Sebastian, Spain) and the Medical Assistance Municipal Institute (Barcelona, Spain). All women gave written informed consent prior to enrollment. Of the 2,150 women who were enrolled during their first trimester of pregnancy, 2,121 completed the occupational questionnaire at 32 weeks of pregnancy, of which 1,739 reported being employed during pregnancy and were followed up to birth (see study flowchart in Fig 1). Of these women, 538 were from Gipuzkoa, 550 from Sabadell, and 651 from Valencia.

### Measurement of fetal growth

Obstetricians performed routine ultrasound examinations at approximately 12, 20, and 34 weeks of pregnancy. At each ultrasound visit, measurements (mm) were recorded for biparietal diameter (BPD), abdominal circumference (AC), and femur length (FL). Based on these parameters, estimated fetal weight (EFW) was calculated [21]. Gestational age was estimated using self-reported last menstrual period or ultrasound data on crown-rump length. The latter was used if gestational age based on the last menstrual period differed from that based on the first ultrasound by at least 7 days.

We used variables representing fetal growth trajectories for INMA participants that were previously modelled as described in Iñiguez et al. [22]. Briefly, using fetal biometry data, linear mixed-effects models were applied to each cohort to obtain longitudinal growth curves for each parameter, i.e., AC, BPD, FL, and EFW. The models were adjusted for constitutional factors known to affect fetal growth: maternal age, height, parity, country of birth (as a proxy of ethnicity), maternal pre-pregnancy weight, father’s height, and fetal sex. From these models, unconditional z-scores at 12, 20 and 34 weeks of gestation were calculated, which represent the deviation in size of a given fetus, in relation to its potential, at the specified time points. Additionally, conditional z-scores, which account for the correlation between repeated measures of a single fetus, were calculated for 12-20 and 20-34 weeks of gestation and represent growth trajectories during the respective time interval [12]. The predicted size of each fetus at each time point was used to calculate the unconditional and conditional z-scores to prevent an increase in random error due to small departures from the regular ultrasound visit schedule. In the present analysis, we focused on the unconditional z-scores at 12 gestational weeks, which represent trajectories in growth from conception up to week 12, or early pregnancy, and the conditional z-scores at 12-20 and 20-34 weeks, which represent growth trajectories during weeks 12-20 (mid-pregnancy) and weeks 20-34 (late pregnancy), respectively.

### Assessment of non-chemical occupational exposures

At approximately 32 weeks of pregnancy, trained interviewers collected information on mothers’ employment status and occupational history. Women who were employed reported the title of their longest held job during the period beginning at least one month pre-conception up to the time of questionnaire administration. Interviewers assessed information on women’s occupational exposure to potential reproductive hazards at the corresponding job. Information on work schedules was collected, including type of shift (fixed vs. rotating shift) and night work (exclusive daytime work vs. any night work). Women also self-reported whether they had no (0 h/day), occasional (<2 h/day), frequent (2-4 h/day), or very frequent (>4 h/day) exposure to physical loads, including standing and lifting objects ≥20 kg. They also reported whether they always, often, sometimes, or never experienced various psychosocial stressors at work. Women who reported often or always experiencing at least three negative psychosocial conditions (i.e., working very fast, requiring constant attention, repercussions of their work on others, having to perform monotonous and repetitive tasks, limited time available to accomplish deadlines, and the possibility of making decisions) and a lack of social support from supervisors and/or coworkers were classified as having exposure to job strain and social isolation [23].

### Assessment of occupational chemical exposures

The job titles of women’s longest held job during pregnancy were linked to International Standard Classification of Occupations (1988 version or ISCO-88, www.ilo.org/public/english/bureau/stat/isco/index.htm) codes. To estimate occupational exposure to EDCs, ISCO88 codes were linked to a job exposure matrix (JEM) created by van Tongeren et al. [24] and later updated Brouwers et al. [25]. This JEM estimates occupational exposure to 10 chemical groups with known or suspected endocrine disrupting properties for which occupational exposure is expected to be significantly higher than exposure from environmental sources. The 10 EDC groups are as follows: polycyclic aromatic hydrocarbons (PAHs), polychlorinated bisphenols (PCBs), pesticides, phthalates, organic solvents, bisphenol A (BPA), APCs, brominated flame retardants (BFRs), metals and miscellaneous (benzophenones, parabens and siloxanes). Briefly, using literature reviews and prior knowledge, three experts in occupational hygiene estimated exposure probability scores for each chemical group for 353 job titles. The exposure probability scores refer to exposure levels that are expected to exceed background levels in the general population and were categorized into three levels: “unlikely” (unlikely to exceed general background concentrations) “possible” (expected to occur among 10% of workers with a given job title) or “probable” (expected to occur among >10% of workers in a given job title). The JEM also includes a fourth exposure category, “unclassifiable,” which is assigned to job titles that are very broad or non-specific. The JEM makes no distinction between routes of exposure (inhalation, ingestion or dermal).

The JEM utilizes occupations coded according to the Standard Occupational Classification 2000 (SOC2000; https://www.ons.gov.uk/methodology/classificationsandstandards/standardoccupationalclassificationsoc/socarchive). Because job titles of INMA participants were linked to ISCO88 codes, the JEM was first translated from SOC2000 to ISCO88 codes using the CAMSIS tool [26] and expert opinion. Details have been published previously by Birks et al. [11]. Based on mothers’ ISCO88 codes, exposure probably scores were assigned to each woman. A total of 220 (12.7%) women had job codes for which exposure was assigned as “unclassifiable.” As a result, exposure probability scores were assigned to 1,519 women. Because data in the “probable” category were sparse, we collapsed the “possible” and “probable” exposure categories to create a dichotomous exposure variable for each of the 10 EDC groups.

### Covariates

The following variables were obtained from the first trimester questionnaire (approximately 12 weeks of gestation): maternal age at conception (years), highest achieved educational level (up to primary, secondary and university), country of birth (Spain vs. foreign), pre-pregnancy weight, height, and parity (0, 1 or ≥ 2 previous pregnancies). Using maternal height and self-reported weight information, pre-pregnancy body mass index (BMI; kg/m^2^) was calculated and classified as underweight, normal weight, overweight or obese. Gestational weight gain was classified as recommended, low or high following the Institute of Medicine (IOM) guidelines [27]. Women were categorized as having smoked if they reported any active smoking during pregnancy at the first and/or third trimester questionnaire (any active smoking during pregnancy, yes vs. no). Maternal alcohol consumption (at least one drink per week, yes vs. no) was obtained from the third trimester questionnaire.

### Statistical analysis

To account for potential heterogeneity in the association between exposure and response variables between INMA regions (i.e., Gipuzkoa, Sabadell, Valencia), we applied mixed effect models to assess the association between occupational exposures and z-scores representing fetal growth during different stages of pregnancy (0-12, 12-20 and 20-34 gestational weeks), treating the exposure variables as fixed effects and including a random intercept to account for clustering of subjects within region. We created a directed acyclic graph (DAG) as a conceptual model for the association between occupational exposures and fetal growth and to guide the selection of covariates in the model. Based on the DAG, we included all the variables listed in the previous paragraph. In models examining EDC exposures, we additionally adjusted for shift work (fixed vs. rotating work shifts). In all models, we treated the exposure of interest independently, i.e., we did not mutually adjust for other exposure variables.

To facilitate comparison between fetal parameters, we converted model coefficients and confidence intervals from z-scores to the scale of the fetal growth parameter consistent with the methodology used Iñiguez et al. [22]. Using fetal biometry data, we converted model coefficients by multiplying by the standard deviation of the respective fetal parameter at each time point (i.e., 12, 20, or 34 gestational weeks) and dividing by their respective means. After multiplying by 100, we can interpret the converted model coefficient as the percent change in the fetal parameter relative to the INMA population mean, in the exposed group compared to the unexposed group.

We performed statistical analyses using SAS 9.4 (Cary, NC) and considered associations with *p*-values < 0.05 as statistically significant.

## Results

Women were, on average, approximately 30 years of age at delivery (Table 1). Most of the women were nulliparous (58.9%) and had secondary or university education (75.6%; see Table 1). Women most frequently reported experiencing standing for a long duration (58.8%) or job strain (23.3%) at their workplaces (see Table 2). Almost one-fourth (26.9%) of women were classified as occupationally exposed to any EDC group, with organic solvents and APCs as the most prevalent exposures (19.2% and 15.9%, respectively). Exposure to the following EDCs were not considered in our analysis because fewer than 1% of women were classified as occupationally exposed to them: polychlorinated organic compounds, bisphenol A and brominated flame retardants (see Table 2). The distribution of the 3-level exposure variables (i.e., “possible,” “probable,” and “unlikely”) is summarized in S1 Table.

**Table 1:** Distribution of maternal characteristics, INMA, 2003-2008 (N = 1,739).

Maternal characteristics	
Age at delivery (years), mean ± SD	30.4 ± 4.1
Educational attainment, *n* (%)	
Primary	368 (24.2)
Secondary	600 (39.5)
University	549 (36.1)
Missing	2 (0.1)
Country of birth, *n* (%)	
Spain	1385 (91.2)
Other	131 (8.6)
Missing	3 (0.2)
Gestational weight gain, *n* (%) ^a^	
Recommended	547 (36.0)
Low	345 (22.7)
High	569 (37.5)
Missing	58 (3.8)
Pre-pregnancy body mass index (BMI, kg/m^3^), *n* (%)	
Underweight (BMI < 18.5)	69 (4.5)
Normal weight (18.5 ≤ BMI < 25)	1077 (70.9)
Overweight (25 ≤ BMI < 30)	272 (17.9)
Obese (≥ 30)	101 (6.6)
Parity, *n* (%)	
0	894 (58.9)
1	549 (36.1)
≥2	74 (4.9)
Missing	2 (0.1)
Smoking during pregnancy, *n* (%) ^b^	
No	1000 (65.8)
Yes	492 (32.4)
Missing	27 (1.8)
Alcohol use during pregnancy, *n* (%) ^c^	
No	1336 (88.0)
Yes	144 (9.5)
Missing	39 (2.6)

SD, standard deviation.

^a^ Gestational weight gain classified according to Institute of Medicine (IOM) guidelines [IOM/NCR (2009)].

^b^ Self-reported maternal active smoking (yes/no) at 12 and/or 32 weeks of pregnancy.

^c^ At least one drink per week during pregnancy (self-reported at 32 weeks of pregnancy).

**Table 2:** Prevalence [*n* (%)] of self-reported non-chemical occupational exposures and estimated exposure to endocrine disrupting chemicals during pregnancy, INMA, 2003-2008.

**Self-reported non-chemical exposures (N = 1,739)**	**n (%)**
Standing **^a^**	1022 (58.8)
Heavy lifting (≥ 20 kg) **^a^**	124 (7.1)
Rotating shift work	136 (7.8)
Any night work	198 (11.4)
Job strain and social isolation ^b^	404 (23.2)
**Estimated EDC exposure (N = 1,519)** **^c^**^,^**^d^**	**n (%)**
PAHs	68 (4.5)
Polychlorinated organic compounds	6 (0.4)
Pesticides	18 (1.2)
Phthalates	63 (4.1)
Organic solvents	291 (19.2)
Bisphenol A	10 (0.7)
APCs	242 (15.9)
Brominated flame retardants	13 (0.9)
Metals	70 (4.6)
Miscellaneous ^e^	52 (3.4)

EDC: Endocrine disrupting chemical; PAHs: Polycyclic aromatic hydrocarbons; APCs: Alkylphenolic compounds; Missing exposure data: Standing, 2.2%; Heavy lifting (≥ 20 kg), 24.7%; Rotating shift work, 2.4%; Any night work, 3.6%; Job strain, 2.5%.

^a^ Frequent (2-4 h day) or very frequent (>4 h day) exposure.

^b^ Self-reported exposure to at least 3 psychosocial conditions and at least one lack of support condition.

^c^ EDC exposure estimated using job-exposure matrix developed by Brouwers et al. (2009).

^d^ n = 220 women with “unclassifiable” EDC exposure score.

^e^ Miscellaneous chemicals include benzophenones, parabens, and siloxanes.

Most associations between non-chemical occupational stressors and fetal growth trajectories were null, with few exceptions. Regarding specific fetal parameters, frequent heavy lifting (≥20 kg) was significantly associated with decreased FL growth between 20-34 weeks (-0.9% [95% confidence interval (CI): -1.6, -0.2%]) but not with BPD or AC (Table 3). We observed an association of similar magnitude between heavy lifting (≥20 kg) and EFW trajectories during the same time period (20-34 weeks: -1.1% (-2.7, 0.4%), although this association was not statistically significant. Night work and rotating shift work were each positively associated with EFW trajectories in mid-pregnancy (i.e., 12-20 weeks; 1.6% [-0.4, 3.6%]) and 1.0% [-0.7, 2.7%], respectively), although these associations were also not statistically significant (Table 3). When considering the percent change in unconditional z-scores at 20 and 34 weeks of pregnancy, the pattern of results was similar to the conditional z-scores (s3 Table).

**Table 3:** Associations between self-reported exposure to non-chemical occupational factors and growth trajectories in estimated fetal weight, INMA, 2003-2008.

			Fetal growth trajectories		
			0-12 weeks	12-20 weeks	20-34 weeks
* *	N	n exposed	% change (95% CI)	% change (95% CI)	% change (95% CI)
**Estimated fetal weight (EFW)**					
Standing **^a^**	1,593	972	0.3% (-0.8, 1.5%)	0.2% (-1.3, 1.0%)	-0.1% (-1.0, 0.7%)
Heavy lifting (≥ 20 kg) **^a^**	1,213	111	-1.0% (-3.0, 1.1%)	-0.5% (-2.6, 1.6%)	-1.1% (-2.7, 0.4%)
Rotating shifts ^b^	1,574	186	0.6% (-1.0, 2.2%)	1.0% (-0.7, 2.7%)	0.6% (-0.8, 1.8%)
Any night work	1,576	125	0.3% (-1.6, 2.2%)	1.6% (-0.4, 3.6%)	0.6% (-0.8, 2.0%)
Job strain and social isolation ^c^	1,589	381	0.3% (-0.9, 1.6%)	-0.9% (-2.2, 0.3%)	0.7% (-0.2, 1.6%)
**Biparietal diameter (BPD)**					
Standing **^a^**	1,598	976	0.1% (-0.9%, 1.1%)	0.4% (-0.1%, 0.9%)	-0.2% (-0.5%, 0.1%)
Heavy lifting (≥ 20 kg) **^a^**	1,217	112	-0.8% (-2.7%, 1.1%)	-0.4% (-1.3%, 0.5%)	-0.1% (-0.7%, 0.5%)
Rotating shifts ^b^	1,579	187	0.5% (-0.9%, 2.0%)	0.1% (-0.6%, 0.8%)	0.0% (-0.4%, 0.5%)
Any night work	1,581	126	0.6% (-1.1%, 2.3%)	0.2% (-0.7%, 1.0%)	0.0% (-0.6%, 0.5%)
Job strain and social isolation ^c^	1,594	383	-0.2% (-1.3%, 0.9%)	0.0% (-0.5%, 0.6%)	0.0% (-0.4%, 0.3%)
**Abdominal circumference (AC)**					
Standing **^a^**	1,600	977	0.3% (-0.7%, 1.2%)	-0.2% (-0.7%, 0.3%)	0.1% (-0.2%, 0.5%)
Heavy lifting (≥ 20 kg) **^a^**	1,217	112	-0.4% (-2.2%, 1.4%)	0.1% (-0.8%, 1.1%)	-0.3% (-1.0%, 0.4%)
Rotating shifts ^b^	1,581	187	0.8% (-0.6%, 2.2%)	0.4% (-0.4%, 1.1%)	0.5% (0.0%, 1.0%)
Any night work	1,583	126	0.5% (-1.2%, 2.1%)	0.5% (-0.4%, 1.4%)	0.3% (-0.3%, 0.9%)
Job strain and social isolation ^c^	1,596	384	0.2% (-0.8%, 1.3%)	-0.3% (-0.8%, 0.3%)	0.3% (-0.1%, 0.7%)
**Femur length (FL)**					
Standing **^a^**	1,599	976	0.4% (-2.0%, 2.7%)	0.1% (-0.5%, 0.6%)	-0.2% (-0.5%, 0.2%)
Heavy lifting (≥ 20 kg) **^a^**	1,218	112	-1.3% (-5.5%, 3.0%)	-0.8% (-1.9%, 0.3%)	-0.9% (-1.6%, -0.2%)
Rotating shifts ^b^	1,580	187	0.7% (-2.7%, 4.1%)	0.4% (-0.4%, 1.2%)	-0.2% (-0.7%, 0.3%)
Any night work	1,582	126	-0.8% (-4.8%, 3.3%)	0.7% (-0.3%, 1.7%)	0.1% (-0.5%, 0.7%)
Job strain and social isolation ^c^	1,595	383	0.8% (-1.7%, 3.4%)	-0.4% (-1.0%, 0.3%)	0.2% (-0.2%, 0.5%)

CI: Confidence interval; Models are adjusted for maternal age, country of birth, education, pre-pregnancy BMI, gestational weight gain, smoking during pregnancy, alcohol use during pregnancy and parity.

^a^ Frequent (2-4 h day) or very frequent (>4 h day) exposure compared to infrequent (<2 h day) or no exposure.

^**b**^ Rotating versus fixed shift.

^c^ Self-reported exposure to at least 3 psychosocial conditions and at least one lack of support condition compared to self-reported exposure to <3 psychosocial conditions or no lack of support conditions.

**Fig 2:**
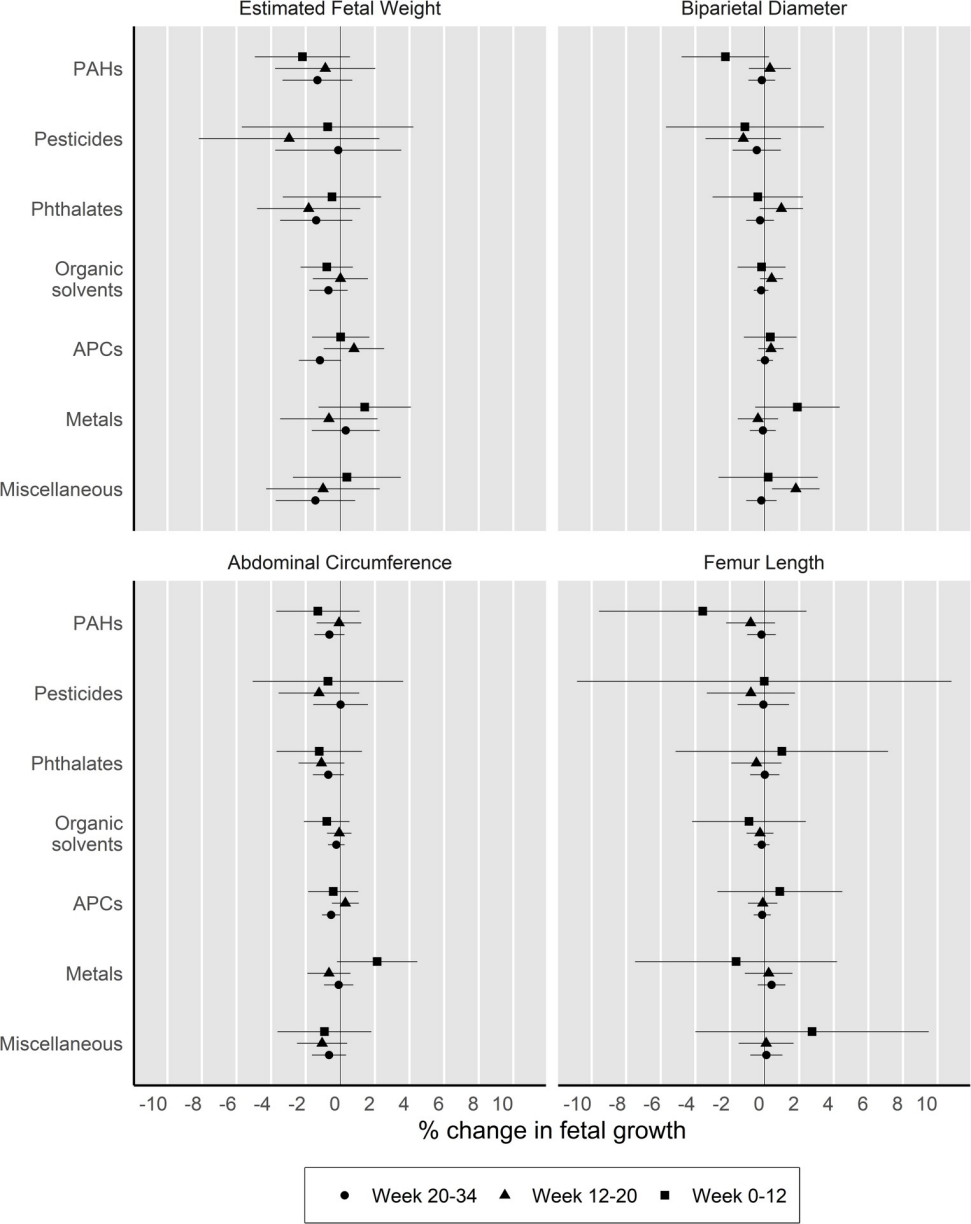
Associations between exposure to endocrine disrupting chemical (EDC) groups and growth trajectories for each fetal parameter, INMA, 2003-2008. **2.** Estimates and their respective 95% confidence intervals are presented as the percentage change in fetal growth compared to participants with unlikely exposure to the respective EDC group at different stages of pregnancy. Models are adjusted for maternal age at birth in years, maternal education, country of birth, gestational weight gain, pre-pregnancy body mass index, active smoking during pregnancy, alcohol consumption during pregnancy, parity and shift work. PAHs: Polycyclic aromatic hydrocarbons, APCs: Alkylphenolic compounds, EFW: Estimated fetal weight (n = 1,403), BPD: Biparietal diameter (n = 1,408), AC: Abdominal circumference (n = 1,410), FL: Femur length (n = 1,409). Numeric estimates are presented in Supplemental Material, S3 Table.

Exposure to phthalates, APCs and miscellaneous chemicals (i.e., benzophenones, parabens and siloxanes) were consistently associated with decreased growth trajectories in EFW and/or AC during late pregnancy, although not all associations were statistically significant (Fig 2). Specifically, we observed associations of similar magnitude between each of these chemical groups and decreased trajectories in EFW during 20-34 weeks of pregnancy (phthalates: -1.4% [-3.5, 0.6%]; APCs -1.1% [-2.3, 0.1%]; miscellaneous chemicals: -1.5% [-3.7, 0.8%]). Reduced trajectories in AC during the same time period were significantly associated with potential exposure to APCs (-0.5% [-1.0, 0.0%]), and while not statistically significant, we observed associations of comparable magnitude with potential exposure to phthalates (-0.6% [-1.5, 0.2%]) and miscellaneous chemicals (-0.6% [-1.6, 0.4%]). We also observed associations between exposure to specific EDC groups and growth trajectories that were in the opposite direction than expected. Exposure to miscellaneous chemical and phthalates were each significantly associated with increased BDP trajectories during mid-pregnancy (12-20 weeks: 2.1% [0.8, 3.5%] and 1.3% [0.1, 2.5%], respectively). Exposure to metals was associated with an increase of similar magnitude in fetal growth trajectories in EFW (1.9% [-0.8, 4.5%]), BPD (2.1% [-0.3, 4.5%]) and AC (2.6% [0.3, 4.9%]) between conception and 12 weeks of gestation. Lastly, we did not find evidence of associations between exposure to EDC groups and FL. Overall, the pattern of results for unconditional z-scores at 20 and 34 weeks was similar to the conditional z-scores (S3 Table).

In an ad hoc analysis, we examined the distribution of job titles among women with possible or probable exposure to EDC groups. The majority of women classified as exposed to phthalates and miscellaneous chemicals worked as hairdressers (67% and 81%, respectively; S4 Table). Of women with exposure to APCs, most were employed as domestic cleaners or hairdressers (68% and 18%, respectively; S4 Table).

## Discussion

We found only limited evidence that occupational exposures to EDCs and non-chemical hazards affect ultrasound-measured fetal growth trajectories. We observed consistent but statistically non-significant associations between phthalates, APCs and miscellaneous chemicals and reduced fetal growth trajectories in late pregnancy. Notably, hairdressers and domestic cleaners accounted for the majority of women classified as exposed to phthalates, APCs and/or miscellaneous chemicals (i.e., benzophenones, parabens and siloxanes). For most job-related non-chemical stressors, we found no evidence of an association with fetal growth trajectories.

The potential mechanism of action by which strenuous physical activities may disrupt fetal growth is unclear but could involve the reduction of uterine blood flow and increased intrauterine pressure, which if continued over a prolonged duration, could affect nutrient availability for the fetus [28,29]. A recent meta-analysis demonstrated that work-related prolonged standing and heavy lifting may be associated with a low to moderate risk of reduced measures of size at birth [16]. Our study finds limited evidence of an association with ultrasound-based measures of fetal growth, which agrees with another study by Snijder et al. [19] that examined similar exposures and ultrasound-based measures of fetal growth among pregnant women in the Netherlands. However, there are a few inconsistent findings. For instance, we observed no impact of standing for a long duration on fetal growth, while Snijder et al. [19] observed decreased head circumference at 30 weeks of pregnancy associated with prolonged standing at work (-0.72 mm [95% CI: -1.43, -0.01]). The authors of this study also examined frequent heavy lifting (≥25 kg) at work but found no association with fetal outcomes, while we observed reduced FL trajectories associated with frequent lifting of objects ≥20 kg. The conflicting results may be attributable to different characteristics of the study populations or differences in fetal growth measures.

The current literature suggests a negative impact of shift work and work-related psychosocial stress on measures of size at birth, but evidence in inconclusive [17]. To our knowledge, this study is the first to examine shift work and job strain in relation to ultrasound-measured fetal growth. Researchers hypothesize that circadian rhythm disruptions and increased stress that may occur as a result of shift work trigger neuroendocrine responses that could affect fetal growth. For example, prolonged disruptions of circadian rhythms and sleep deprivation may lead to dysregulation of fetal growth hormones (e.g., prolactin) [30,31]. Shift work may also result in increased stress and elevated levels of systemic cortisol, which is associated with alteration in placental growth and function [32]. For women who are employed during pregnancy, occupational stress is common and may be an important contributor to overall psychosocial stress. Further, unlike most stressful events in individuals’ personal lives, psychosocial stress arising from work conditions is potentially preventable.

The process of fetal growth is carefully orchestrated by endocrine factors. Prenatal exposure to EDCs may affect the fetus directly or dysregulate placental function via several hormonal pathways, including disruptions in thyroid or insulin hormone homeostasis as well as epigenetic and inflammatory pathways, with negative consequences for fetal growth [33,34]. Previous epidemiologic studies that have utilized the same JEM employed in our analysis report associations between maternal occupational exposure to EDCs and anthropometric measures of size at birth [9–11]. For example, Birks et al. [11] observed increased odds of LBW in relation to maternal exposures to PAHs (OR = 1.62, 95% CI: 1.12-2.34), pesticides (1.85, 1.15-2.98), phthalates (2.35, 1.16-4.75), APCs (1.33, 1.02-1.74), brominated flame retardants (3.88, 1.37-11.02) and metals (1.53, 1.13-2.07). In our analysis examining measures of growth across the prenatal period, we found limited evidence for a role of JEM-based EDC exposures on fetal growth. Nevertheless, noticeable patterns arose among growth trajectories for specific body segments associated with specific EDC groups. For example, we observed associations of exposure to APCs with reduced growth in EFW and AC during late pregnancy. One other study has utilized Brouwer’s JEM to evaluate the potential role of maternal EDC exposures in ultrasound measures of fetal growth. Among a birth cohort in the Netherlands, Snijder et al. [14] also found that JEM-based APC exposure was associated with reduced growth in EFW, although this was not a statistically significant finding. The authors did, however, report a significant association between APCs and decreased trajectories in head circumference (β = -0.018, with beta coefficients representing the average weekly decline in EFW standard deviation [SD]-scores). Additionally, the authors found significant negative associations of exposure to PAHs and phthalates with EFW SD-scores, while our results regarding PAHs and EFW trajectories in late pregnancy were similar in direction but not statistically significant. While Snijder et al. [14] estimated the average change in SD-scores per gestational week, our analysis estimated the average change in z-scores within specified periods of gestation. Given that we might not expect fetal growth trajectories to be linear over time, it may be more appropriate to examine average trajectories during windows of pregnancy defined *a priori*. In addition, Snijder et al. [14] only evaluated fetal growth trajectories from the second trimester (i.e., 18-25 weeks of gestation) onward. In our analysis, we examined growth trajectories during early and mid-pregnancy (i.e., 0-12 and 12-20 weeks). Considering growth trajectories in early pregnancy, we found positive associations with exposure to metals and negative associations with exposure to PAHs (though not statistically significant). We also observed increased BPD trajectories in mid-pregnancy associated with occupational exposure to miscellaneous chemicals.

Among women in our study, 26.9% were classified exposed to any EDC group, which is higher than what has been reported in other European birth cohort studies that classify exposure using the same JEM employed in our study. For example, Snijder et al. [14] reported the prevalence of exposure to any EDC group in their study population as 6.7%, and a meta-analysis of 13 European birth cohorts reported the overall prevalence as 11.4% [11]. In our study, the high exposure prevalence is driven by exposures to organic solvents and APCs (19.2% and 15.9%, respectively). As mentioned previously, domestic cleaners and hairdressers make up the majority of women classified as exposed to these compounds, and notably, there are fewer women employed in these two occupations among women in birth cohorts located in Northern European countries [35]. However, our study is comparable in this regard to other Southern European birth cohorts, including Generation XXI (Portugal), INMA-Granada (Spain), and Rhea (Greece), which is reflected in the higher prevalence of exposure to organic solvents and APCs among women in their study populations [35]. Thus, our study findings may not be generalizable beyond Southern European populations.

As mentioned previously, the majority of women classified as exposed to phthalates, APCs, and miscellaneous chemicals were employed as hairdressers or domestic cleaners. Therefore, it is difficult to determine whether the findings of this study are in fact attributable to the exposure of interest or confounded by co-occurring exposures or by other unmeasured job-related exposures. For example, hairdressers may be exposed to other chemicals not evaluated in this study through the use of hair products such as hair spray, dyes and shampoos. Similarly, domestic cleaners may be exposed to other chemical agents in cleaning products that were not evaluated by the JEM. Furthermore, the potential effect of chemical exposures in these occupations may be modified by exposure to non-chemical work stressors such as prolonged standing, irregular work hours, and stress [36]. Further research is needed to understand the potential combined impact of multiple workplace exposures on fetal development.

Our study has important limitations regarding exposure assessment methods. First, the JEM we applied yields crude categories of exposure and assumes homogenous exposure for all members in each category [37]. Therefore, we were unable to account for differences in job tasks and work environments among individuals with the same job title. We also combined the “possible” and “probable” exposure categories for each EDC group, potentially introducing additional exposure misclassification. Although the JEM has not been validated, it has been widely used in the literature [9–11,14], including in the INMA project and other European birth cohorts, enabling comparison with other studies. While most women are likely to have some level of exposure to EDCs through diet and consumer products, we do not have reason to suspect that background exposure is associated with exposures in individual occupations and thus should not confound the observed associations. For non-chemical occupational stressors, exposure was self-reported and thus subject to error in recall. However, we expect any resulting exposure misclassification to be non-differential. Given the large number of associations assessed in our study, it is possible that we observed some associations due to chance. Lastly, due to the low estimated exposure prevalence of many EDC groups, our study was not adequately powered to examine the cumulative or interactive effect of co-occurring chemical and non-chemical stressors.

The greatest advantage of our study is the use of ultrasound-based measures of fetal growth. Compared with birth anthropometry, the use of statistical methods to compare observed and expected growth potential for each fetus reduces potential for misclassification, because such methods allow us to discriminate between constitutionally small versus pathologically small fetuses. Further, utilizing z-scores to quantify trimester-specific fetal growth trajectories grants the potential to gain insight into windows of developmental susceptibility. Another strength of this analysis is the availability of detailed information on several individual-level maternal factors, allowing us to minimize potential confounding.

## Conclusions

Knowledge of the developmental impacts of most occupational hazards is incomplete [38]. Given that in Western countries, most women will work during their childbearing years, it is important to understand whether occupational hazards increase the risk of adverse consequences on fetal development in order to protect the health of future generations. This study finds limited evidence of an effect of maternal occupational exposures on ultrasound-based measures of fetal growth but reported specific occupational groups that have a relatively large burden of work-related EDC exposure. Our study supports the need for further research that considers the cumulative or combined impact of work-related chemical and non-chemical stressors among pregnant women.

## Supporting information

S1 Table Distribution of 3-level exposure categories estimated using a job exposure matrix, INMA, 2003-2008 (N = 1,519). (DOCX)

S2 Table Percent change in fetal growth from 0-20 and 0-34 weeks of pregnancy and their respective 95% confidence intervals associated with self-reported exposure to non-chemical occupational stressors, INMA, 2003-2008. (DOCX)

S3 Table Percent change in fetal growth and their respective 95% confidence intervals at different stages of pregnancy associated with occupational endocrine disrupting chemicals (EDC) exposure, INMA, 2003-2008. (DOCX)

S4 Table Distribution of job titles among women with possible or probable exposure to endocrine disrupting chemical (EDC) groups as classified by a job-exposure matrix, INMA, 2003-2006 (N = 409). (DOCX)
