# Supplementary material for: Maternal occupational exposures and fetal growth in a Spanish birth cohort
Source: PLoS One. 2022 Apr 7;17(4):e0264530. doi: 10.1371/journal.pone.0264530 (PMC8989310; doi:10.1371/journal.pone.0264530)
Supplement: S1 Table — (DOCX) [file pone.0264530.s001.docx]

**S1 Table. Distribution of 3-level exposure categories for each endocrine disrupting chemical group estimated using a job exposure matrix, by cohort, INMA, 2003-2008 (N = 1,519)**

| **EDC Exposure** | **n (%)** |
| --- | --- |
| Polycyclic aromatic hydrocarbons |  |
| Unlikely | 1451 (95.5%) |
| Possible | 9 (0.6%) |
| Probable | 59 (3.9%) |
| Polychlorinated organic compounds |  |
| Unlikely | 1513 (99.6%) |
| Possible | 6 (0.4%) |
| Probable | 0 (0.0%) |
| Pesticides |  |
| Unlikely | 1501 (98.8%) |
| Possible | 12 (0.8%) |
| Probable | 6 (0.4%) |
| Phthalates |  |
| Unlikely | 1455 (95.8%) |
| Possible | 8 (0.5%) |
| Probable | 56 (3.7%) |
| Organic solvents |  |
| Unlikely | 1227 (80.8%) |
| Possible | 269 (17.7%) |
| Probable | 23 (1.5%) |
| Bisphenol A |  |
| Unlikely | 1509 (99.3%) |
| Possible | 10 (0.7%) |
| Probable | 0 (0.0%) |
| Alkylphenolic compounds |  |
| Unlikely | 1276 (84.0%) |
| Possible | 243 (16.0%) |
| Probable | 0 (0.0%) |
| Brominated flame retardants |  |
| Unlikely | 1506 (99.1%) |
| Possible | 13 (0.9%) |
| Probable | 0 (0.0%) |
| Metals |  |
| Unlikely | 1449 (95.4%) |
| Possible | 50 (3.3%) |
| Probable | 20 (1.3%) |
| Miscellaneous |  |
| Unlikely | 1466 (96.5%) |
| Possible | 10 (0.7%) |
| Probable | 43 (2.8%) |

Exposure estimated using job exposure matrix developed by Brouwer’s et al. (2009); Miscellaneous chemicals include benzophenones, parabens, and siloxanes.
