# Supplementary material for: Maternal occupational exposures and fetal growth in a Spanish birth cohort
Source: PLoS One. 2022 Apr 7;17(4):e0264530. doi: 10.1371/journal.pone.0264530 (PMC8989310; doi:10.1371/journal.pone.0264530)
Supplement: S2 Table — (DOCX) [file pone.0264530.s002.docx]

**S2 Table. Percent change in fetal growth from 0-20 and 0-34 weeks of pregnancy and their respective 95% confidence intervals associated with self-reported exposure to non-chemical occupational stressors, INMA, 2003-2008**

|  |  |  | **Unconditional z-scores** | |
| --- | --- | --- | --- | --- |
|  |  |  | **0-20 weeks** | **0-34 weeks** |
|  | N | N exposed | % change (95% CI) | % change (95% CI) |
| **Estimated fetal weight** |  |  |  |  |
| Standing ^a^ | 1,593 | 972 | 0.0% (-1.1%, 1.2%) | -0.1% (-1.0%, 0.7%) |
| Heavy lifting (≥ 20 kg) ^a^ | 1,213 | 111 | -0.9% (-3.0%, 1.3%) | -1.1% (-2.7%, 0.4%) |
| Rotating shift work ^b^ | 1,574 | 186 | 1.2% (-0.5%, 2.9%) | 0.6% (-0.6%, 1.8%) |
| Any night work | 1,576 | 125 | 1.5% (-0.5%, 3.6%) | 0.6% (-0.8%, 2.0%) |
| Job strain + social isolation ^c^ | 1,589 | 381 | -0.6% (-1.9%, 0.7%) | 0.4% (-0.5%, 1.4%) |
| **Biparietal diameter** |  |  |  |  |
| Standing ^a^ | 1,598 | 976 | 0.4% (-0.1%, 0.9%) | -0.1% (-0.4%, 0.2%) |
| Heavy lifting (≥ 20 kg) ^a^ | 1,217 | 112 | -0.5% (-1.4%, 0.3%) | -0.2% (-0.8%, 0.4%) |
| Rotating shift work ^b^ | 1,579 | 187 | 0.2% (-0.5%, 0.9%) | 0.1% (-0.4%, 0.5%) |
| Any night work | 1,581 | 126 | 0.3% (-0.5%, 1.1%) | 0.0% (-0.5%, 0.6%) |
| Job strain + social isolation ^c^ | 1,594 | 383 | 0.0% (-0.5%, 0.5%) | 0.0% (-0.4%, 0.3%) |
| **Abdominal circumference** |  |  |  |  |
| Standing ^a^ | 1,600 | 977 | -0.1% (-0.6%, 0.4%) | 0.1% (-0.3%, 0.4%) |
| Heavy lifting (≥ 20 kg) ^a^ | 1,217 | 112 | 0.0% (-0.9%, 1.0%) | -0.3% (-1.0%, 0.4%) |
| Rotating shift work ^b^ | 1,581 | 187 | 0.5% (-0.2%, 1.3%) | 0.6% (0.0%, 1.1%) |
| Any night work | 1,583 | 126 | 0.6% (-0.3%, 1.5%) | 0.4% (-0.2%, 1.0%) |
| Job strain + social isolation ^c^ | 1,596 | 384 | -0.2% (-0.7%, 0.4%) | 0.2% (-0.2%, 0.6%) |
| **Femur length** |  |  |  |  |
| Standing ^a^ | 1,599 | 976 | 0.1% (-0.4%, 0.7%) | -0.2% (-0.5%, 0.2%) |
| Heavy lifting (≥ 20 kg) ^a^ | 1,218 | 112 | -1.0% (-2.0%, 0.1%) | -1.0% (-1.7%, -0.3%) |
| Rotating shift work ^b^ | 1,580 | 187 | 0.4% (-0.4%, 1.2%) | -0.1% (-0.6%, 0.4%) |
| Any night work | 1,582 | 126 | 0.5% (-0.5%, 1.4%) | 0.1% (-0.5%, 0.7%) |
| Job strain + social isolation ^c^ | 1,595 | 383 | -0.2% (-0.8%, 0.4%) | 0.0% (-0.3%, 0.5%) |

All models are adjusted for maternal age, country of birth, education, pre-pregnancy body mass index, gestational weight gain, smoking during pregnancy, alcohol use during pregnancy and parity

^a^ Frequent (2-4 h day) or very frequent (>4 h day) exposure compared to occasional (<2 h day) or no exposure

^b^ Fixed vs. rotating shifts

^c^ self-reported exposure to at least 3 psychosocial conditions and at least one lack of support condition
