# Supplementary material for: Maternal occupational exposures and fetal growth in a Spanish birth cohort
Source: PLoS One. 2022 Apr 7;17(4):e0264530. doi: 10.1371/journal.pone.0264530 (PMC8989310; doi:10.1371/journal.pone.0264530)
Supplement: S3 Table — (DOCX) [file pone.0264530.s003.docx]

**S3 Table. Percent change in fetal growth and their respective 95% confidence intervals at different stages of pregnancy associated with occupational endocrine disrupting chemical (EDC) exposure, INMA, 2003-2008**

|  | **Unconditional z-scores** | | | **Conditional z-scores** | |
| --- | --- | --- | --- | --- | --- |
|  | **12 weeks** | **20 weeks** | **34 weeks** | **12-20 weeks** | **20-34 weeks** |
|  | % change (95% CI) | % change (95% CI) | % change (95% CI) | % change (95% CI) | % change (95% CI) |
| **Estimated fetal weight (n = 1,403)** |  |  |  |  |  |
| Polycyclic aromatic hydrocarbons | -2.1% (-4.7%, 0.6%) | -2.0% (-4.9%, 0.8%) | -1.3% (-3.3%, 0.8%) | -1.1% (-3.9%, 1.7%) | -0.7% (-2.6%, 1.3%) |
| Pesticides | -0.7% (-5.7%, 4.2%) | -2.8% (-8.2%, 2.6%) | -1.0% (-4.8%, 2.8%) | -2.9% (-8.2%, 2.3%) | -0.2% (-3.9%, 3.5%) |
| Phthalates | 0.1% (-2.7%, 2.9%) | -0.6% (-3.6%, 2.5%) | -1.4% (-3.6%, 0.7%) | -0.8% (-3.7%, 2.2%) | -1.4% (-3.5%, 0.6%) |
| Organic solvents | -0.6% (-2.1%, 0.9%) | -0.4% (-2.0%, 1.2%) | -0.7% (-1.9%, 0.4%) | -0.1% (-1.6%, 1.5%) | -0.7% (-1.8%, 0.4%) |
| Alkylphenolic compounds | 0.2% (-1.4%, 1.8%) | 0.7% (-1.1%, 2.5%) | -0.8% (-2.1%, 0.4%) | 0.7% (-1%, 2.5%) | -1.1% (-2.3%, 0.1%) |
| Metals | 1.9% (-0.8%, 4.5%) | 0.9% (-2.0%, 3.7%) | 0.5% (-1.6%, 2.5%) | -0.2% (-3%, 2.6%) | 0.3% (-1.6%, 2.3%) |
| Miscellaneous | 1.1% (-2.0%, 4.1%) | 0.8% (-2.5%, 4.1%) | -1.1% (-3.4%, 1.3%) | 0.2% (-3%, 3.4%) | -1.5% (-3.7%, 0.8%) |
| **Biparietal diameter (n = 1,408)** | |  |  |  |  |
| Polycyclic aromatic hydrocarbons | -2.0% (-4.4%, 0.5%) | -0.3% (-1.4%, 0.8%) | -0.2% (-1.0%, 0.5%) | 0.2% (-1.0%, 1.4%) | -0.2% (-0.9%, 0.6%) |
| Pesticides | -1.2% (-5.7%, 3.4%) | -1.3% (-3.4%, 0.8%) | -0.7% (-2.1%, 0.7%) | -1.2% (-3.4%, 1.0%) | -0.5% (-1.8%, 0.9%) |
| Phthalates | -0.1% (-2.6%, 2.5%) | 1.1% (-0.1%, 2.3%) | 0.0% (-0.8%, 0.8%) | 1.3% (0.1%, 2.5%) | -0.2% (-1.0%, 0.5%) |
| Organic solvents | -0.1% (-1.4%, 1.3%) | 0.4% (-0.2%, 1%) | -0.1% (-0.5%, 0.3%) | 0.5% (-0.2%, 1.1%) | -0.2% (-0.6%, 0.2%) |
| Alkylphenolic compounds | 0.4% (-1.1%, 1.9%) | 0.5% (-0.2%, 1.2%) | 0.1% (-0.3%, 0.6%) | 0.4% (-0.3%, 1.2%) | 0.0% (-0.4%, 0.5%) |
| Metals | 2.1% (-0.3%, 4.5%) | 0.3% (-0.8%, 1.4%) | -0.1% (-0.8%, 0.7%) | -0.2% (-1.4%, 0.9%) | -0.1% (-0.9%, 0.6%) |
| Miscellaneous | 0.6% (-2.2%, 3.4%) | 2.0% (0.7%, 3.3%) | 0.2% (-0.6%, 1.1%) | 2.1% (0.8%, 3.5%) | -0.2% (-1.0%, 0.7%) |
| **Abdominal circumference (n = 1,410)** | |  |  |  |  |
| Polycyclic aromatic hydrocarbons | -0.9% (-3.2%, 1.4%) | -0.5% (-1.8%, 0.7%) | -0.5% (-1.3%, 0.4%) | -0.3% (-1.5%, 1.0%) | -0.4% (-1.3%, 0.4%) |
| Pesticides | -0.8% (-5.2%, 3.6%) | -1.3% (-3.6%, 1.0%) | -0.3% (-1.9%, 1.3%) | -1.2% (-3.5%, 1.1%) | 0.0% (-1.6%, 1.6%) |
| Phthalates | -0.5% (-3.0%, 1.9%) | -0.7% (-2.0%, 0.6%) | -0.7% (-1.6%, 0.2%) | -0.7% (-2.0%, 0.6%) | -0.6% (-1.5%, 0.2%) |
| Organic solvents | -0.6% (-1.9%, 0.7%) | -0.2% (-0.9%, 0.5%) | -0.2% (-0.7%, 0.3%) | -0.1% (-0.8%, 0.6%) | -0.2% (-0.7%, 0.3%) |
| Alkylphenolic compounds | -0.3% (-1.7%, 1.2%) | 0.2% (-0.6%, 1.0%) | -0.4% (-0.9%, 0.1%) | 0.3% (-0.5%, 1.1%) | -0.5% (-1.0%, 0.0%) |
| Metals | 2.6% (0.3%, 4.9%) | 0.2% (-1.0%, 1.4%) | 0% (-0.8%, 0.9%) | -0.5% (-1.8%, 0.7%) | 0.0% (-0.8%, 0.8%) |
| Miscellaneous | -0.1% (-2.7%, 2.6%) | -0.5% (-1.9%, 0.9%) | -0.6% (-1.6%, 0.3%) | -0.6% (-2.0%, 0.9%) | -0.6% (-1.6%, 0.4%) |
| **Femur length (n = 1,409)** |  |  |  |  |  |
| Polycyclic aromatic hydrocarbons | -4.2% (-9.9%, 1.6%) | -1.0% (-2.4%, 0.3%) | -0.2% (-1.0%, 0.6%) | -0.6% (-2.0%, 0.7%) | 0.0% (-0.8%, 0.8%) |
| Pesticides | 0.0% (-10.8%, 10.9%) | -0.6% (-3.2%, 1.9%) | -0.1% (-1.7%, 1.5%) | -0.8% (-3.4%, 1.8%) | -0.1% (-1.6%, 1.4%) |
| Phthalates | 2.5% (-3.6%, 8.5%) | 0.1% (-1.3%, 1.5%) | 0.0% (-0.9%, 0.9%) | -0.3% (-1.8%, 1.1%) | -0.1% (-0.9%, 0.8%) |
| Organic solvents | -0.4% (-3.7%, 2.8%) | -0.3% (-1.1%, 0.4%) | -0.3% (-0.7%, 0.2%) | -0.3% (-1.1%, 0.4%) | -0.2% (-0.7%, 0.2%) |
| Alkylphenolic compounds | 1.3% (-2.2%, 4.9%) | 0.0% (-0.8%, 0.8%) | -0.1% (-0.7%, 0.4%) | -0.2% (-1.0%, 0.7%) | -0.2% (-0.7%, 0.3%) |
| Metals | -0.3% (-6.0%, 5.5%) | 0.3% (-1%, 1.7%) | 0.3% (-0.5%, 1.1%) | 0.4% (-1.0%, 1.8%) | 0.3% (-0.5%, 1.1%) |
| Miscellaneous | 4.4% (-2.2%, 11.0%) | 0.9% (-0.7%, 2.4%) | 0.2% (-0.8%, 1.1%) | 0.2% (-1.3%, 1.8%) | -0.7% (-2.6%, 1.3%) |

All models are adjusted for maternal age, maternal education, country of birth, pre-pregnancy body mass index, gestational weight gain, smoking during pregnancy, alcohol use during pregnancy, and parity; Reference group: women with unlikely exposure to the respective EDC group
