# Supplementary material for: Maternal occupational exposures and fetal growth in a Spanish birth cohort
Source: PLoS One. 2022 Apr 7;17(4):e0264530. doi: 10.1371/journal.pone.0264530 (PMC8989310; doi:10.1371/journal.pone.0264530)
Supplement: S4 Table — (DOCX) [file pone.0264530.s004.docx]

**S4 Table. Distribution of job titles among women with possible or probable exposure to endocrine disrupting chemical (EDC) groups as classified by a job-exposure matrix, INMA, 2003-2006 (N = 409)**

|  | **PAHs** | **Polychlorinated organic compounds** | **Pesticides** | **Phthalates** | **Organic solvents** |
| --- | --- | --- | --- | --- | --- |
|  | N=68 | N=6 | N=18 | N=64 | N=292 |
| **Job title** |  |  |  |  |  |
| Artists | 0.0% | 0.0% | 0.0% | 0.0% | 0.7% |
| Assemblers (electrical products) | 0.0% | 0.0% | 0.0% | 0.0% | 0.0% |
| Assemblers (vehicles and metal goods) | 0.0% | 0.0% | 0.0% | 0.0% | 5.5% |
| Bookbinders and print finishers | 0.0% | 0.0% | 0.0% | 0.0% | 1.7% |
| Carpenters and joiners | 0.0% | 0.0% | 11.1% | 3.1% | 0.7% |
| Cleaners, domestics | 0.0% | 0.0% | 0.0% | 0.0% | 56.5% |
| Conservation and environmental protection officers | 0.0% | 0.0% | 33.3% | 0.0% | 0.0% |
| Dental practitioners | 0.0% | 0.0% | 0.0% | 0.0% | 0.0% |
| Electrical/electronics technicians | 0.0% | 0.0% | 0.0% | 0.0% | 0.0% |
| Farm workers | 0.0% | 0.0% | 22.2% | 6.2% | 1.4% |
| Fork-lift truck drivers | 2.9% | 0.0% | 0.0% | 0.0% | 0.0% |
| Glass and ceramics makers, decorators and finishers | 0.0% | 0.0% | 0.0% | 0.0% | 1.4% |
| Glass and ceramics process operatives | 0.0% | 0.0% | 0.0% | 0.0% | 0.3% |
| Goldsmiths, silversmiths, precious stone workers | 0.0% | 0.0% | 0.0% | 0.0% | 0.0% |
| Hairdressers, barbers | 0.0% | 0.0% | 0.0% | 67.2% | 14.7% |
| Horticultural trades | 0.0% | 0.0% | 11.1% | 3.1% | 0.7% |
| Laboratory technicians | 0.0% | 0.0% | 0.0% | 0.0% | 1.0% |
| Launderers, dry cleaners, pressers | 0.0% | 0.0% | 0.0% | 0.0% | 0.7% |
| Medical and dental technicians | 0.0% | 0.0% | 0.0% | 0.0% | 0.0% |
| Metal working machine operatives | 0.0% | 0.0% | 0.0% | 0.0% | 4.1% |
| Metal working production and maintenance fitters | 0.0% | 0.0% | 0.0% | 0.0% | 0.3% |
| Moulders, core makers, die casters | 1.5% | 0.0% | 0.0% | 0.0% | 0.0% |
| Painters and decorators | 0.0% | 0.0% | 0.0% | 0.0% | 5.5% |
| Paper and wood machine operatives | 0.0% | 100.0% | 0.0% | 0.0% | 0.0% |
| Paramedics | 4.4% | 0.0% | 0.0% | 0.0% | 0.0% |
| Photographers and audio-visual equipment operators | 0.0% | 0.0% | 0.0% | 0.0% | 0.3% |
| Plastics process operatives | 0.0% | 0.0% | 0.0% | 15.6% | 3.4% |
| Police officers (sergeant and below) | 0.0% | 0.0% | 0.0% | 0.0% | 0.0% |
| Precision instrument makers and repairers | 0.0% | 0.0% | 0.0% | 0.0% | 0.0% |
| Rubber process operatives | 1.5% | 0.0% | 0.0% | 1.6% | 0.3% |
| Screen printers | 0.0% | 0.0% | 0.0% | 3.1% | 0.7% |
| Taxi, cab drivers and chauffeurs | 2.9% | 0.0% | 0.0% | 0.0% | 0.0% |
| Textile process operatives | 0.0% | 0.0% | 0.0% | 0.0% | 0.0% |
| Textiles, garments and related trades | 0.0% | 0.0% | 0.0% | 0.0% | 0.0% |
| Veterinarians | 0.0% | 0.0% | 16.7% | 0.0% | 0.0% |
| Veterinary nurses and assistants | 0.0% | 0.0% | 5.6% | 0.0% | 0.0% |
| Waiters, waitresses | 86.8% | 0.0% | 0.0% | 0.0% | 0.0% |
| Welding trades | 0.0% | 0.0% | 0.0% | 0.0% | 0.0% |

**S4 Table, con’t. Distribution of job titles among women with possible or probable exposure to endocrine disrupting chemical (EDC) groups as classified by a job-exposure matrix, INMA, 2003-2006 (N = 409)**

|  | **Bisphenol A** | **APCs** | **Brominated flame retardants** | **Metals** | **Misc** |
| --- | --- | --- | --- | --- | --- |
|  | N=10 | N=243 | N=13 | N=70 | N=53 |
| **Job title** |  |  |  |  |  |
| Artists | 0.0% | 0.0% | 0.0% | 0.0% | 0.0% |
| Assemblers (electrical products) | 0.0% | 0.0% | 0.0% | 2.9% | 0.0% |
| Assemblers (vehicles and metal goods) | 0.0% | 0.0% | 0.0% | 0.0% | 0.0% |
| Bookbinders and print finishers | 0.0% | 0.0% | 0.0% | 0.0% | 0.0% |
| Carpenters and joiners | 0.0% | 0.0% | 0.0% | 2.9% | 0.0% |
| Cleaners, domestics | 0.0% | 67.9% | 0.0% | 0.0% | 0.0% |
| Conservation and environmental protection officers | 0.0% | 0.0% | 0.0% | 0.0% | 0.0% |
| Dental practitioners | 0.0% | 0.0% | 0.0% | 8.6% | 0.0% |
| Electrical/electronics technicians | 0.0% | 0.0% | 0.0% | 7.1% | 0.0% |
| Farm workers | 0.0% | 1.6% | 0.0% | 5.7% | 0.0% |
| Fork-lift truck drivers | 0.0% | 0.0% | 0.0% | 0.0% | 0.0% |
| Glass and ceramics makers, decorators and finishers | 0.0% | 0.0% | 0.0% | 5.7% | 0.0% |
| Glass and ceramics process operatives | 0.0% | 0.0% | 0.0% | 1.4% | 0.0% |
| Goldsmiths, silversmiths, precious stone workers | 0.0% | 0.0% | 0.0% | 4.3% | 0.0% |
| Hairdressers, barbers | 0.0% | 17.7% | 0.0% | 0.0% | 81.1% |
| Horticultural trades | 0.0% | 0.8% | 0.0% | 2.9% | 0.0% |
| Laboratory technicians | 0.0% | 0.0% | 0.0% | 0.0% | 0.0% |
| Launderers, dry cleaners, pressers | 0.0% | 0.8% | 0.0% | 0.0% | 0.0% |
| Medical and dental technicians | 0.0% | 0.0% | 0.0% | 4.3% | 0.0% |
| Metal working machine operatives | 0.0% | 0.0% | 0.0% | 17.1% | 0.0% |
| Metal working production and maintenance fitters | 0.0% | 0.0% | 0.0% | 1.4% | 0.0% |
| Moulders, core makers, die casters | 0.0% | 0.0% | 0.0% | 0.0% | 0.0% |
| Painters and decorators | 0.0% | 0.0% | 0.0% | 0.0% | 0.0% |
| Paper and wood machine operatives | 0.0% | 2.5% | 0.0% | 0.0% | 0.0% |
| Paramedics | 0.0% | 0.0% | 0.0% | 0.0% | 0.0% |
| Photographers and audio-visual equipment operators | 0.0% | 0.4% | 0.0% | 0.0% | 0.0% |
| Plastics process operatives | 100.0% | 4.1% | 76.9% | 14.3% | 18.9% |
| Police officers (sergeant and below) | 0.0% | 0.0% | 0.0% | 1.4% | 0.0% |
| Precision instrument makers and repairers | 0.0% | 0.0% | 0.0% | 1.4% | 0.0% |
| Rubber process operatives | 0.0% | 0.0% | 7.7% | 0.0% | 0.0% |
| Screen printers | 0.0% | 0.0% | 0.0% | 0.0% | 0.0% |
| Taxi, cab drivers and chauffeurs | 0.0% | 0.0% | 0.0% | 0.0% | 0.0% |
| Textile process operatives | 0.0% | 0.8% | 15.4% | 2.9% | 0.0% |
| Textiles, garments and related trades | 0.0% | 3.3% | 0.0% | 11.4% | 0.0% |
| Veterinarians | 0.0% | 0.0% | 0.0% | 0.0% | 0.0% |
| Veterinary nurses and assistants | 0.0% | 0.0% | 0.0% | 0.0% | 0.0% |
| Waiters, waitresses | 0.0% | 0.0% | 0.0% | 0.0% | 0.0% |
| Welding trades | 0.0% | 0.0% | 0.0% | 4.3% | 0.0% |
